# AI-Powered Embedded System for Rapid Detection of Veterinary Antibiotic Residues in Food-Producing Animals

**DOI:** 10.3390/antibiotics14090917

**Published:** 2025-09-11

**Authors:** Ximing Li, Lanqi Chen, Qianchao Wang, Mengting Zhou, Jingheng Long, Xi Chen, Jiangsan Zhao, Junjun Yu, Yubin Guo

**Affiliations:** 1 College of Mathematics and Informatics, South China Agricultural University, Guangzhou 510642, China; liximing@scau.edu.cn (X.L.); bailanking@stu.scau.edu.cn (L.C.); qianchaowang@stu.scau.edu.cn (Q.W.); 20243170078@stu.scau.edu.cn (J.L.);; 2 State Key Laboratory of Animal Nutrition and Feeding, Institute of Animal Science, Chinese Academy of Agricultural Sciences, Beijing 100193, China; 3 Department of Agricultural Technology, Norwegian Institute of Bioeconomy Research (NIBIO), 2849 Kapp, Norway; 4 Guangdong Enterprise Key Laboratory for Animal Health and Environmental Control, WENS Research Institute (Technology Center), WENS Foodstuff Group Co., Ltd., Yunfu 527400, China

**Keywords:** veterinary antibiotic residue, food safety, object detection, high-throughput, lightweight model, embedded system

## Abstract

**Background:** Veterinary antibiotics are widely used in food-producing animals, raising public health concerns due to drug residues and the risk of antimicrobial resistance. Rapid and reliable detection systems are critical to ensure food safety and regulatory compliance. Colloidal gold immunoassay (CGIA)-based antigen–antibody test cards are widely used in food safety for the rapid screening of veterinary antibiotic residues. However, manual interpretation of test cards remains inefficient and inconsistent. **Methods:** To address this, we propose a complete AI-based detection system for veterinary antibiotic residues. The system is built on the Rockchip RK3568 platform and integrates a five-megapixel OV5640 autofocus USB camera (60° field of view) with a COB LED strip (6000 K, rated 5 W/m). It enables high-throughput, automated interpretation of colloidal gold test cards and can generate structured detection reports for regulatory documentation and quality control. The core challenge lies in achieving accurate and fast inference on resource-constrained embedded devices, where traditional detection networks often struggle to balance model size and performance. To this end, we propose VetStar, a lightweight detection algorithm specifically optimized for this task. VetStar integrates StarBlock, a shallow feature extractor, and Depthwise Separable-Reparameterization Detection Head (DR-head), a compact, partially decoupled detection head that accelerates inference while preserving accuracy. **Results:** Despite its compact size, with only 0.04 M parameters and 0.3 GFLOPs, VetStar maintains strong performance after distillation with the Bridging Cross-task Protocol Inconsistency Knowledge Distillation (BCKD) method. For our custom Veterinary Drug Residue Rapid Test Card (VDR-RTC) dataset, it achieves an mAP50 of 97.4 and anmAP50-95of 89.5. When deployed on the RK3568 device, it delivers results in just 5.4 s—substantially faster than comparable models. **Conclusions:** These results highlight the system’s strong potential for high-throughput, cost-effective, and rapid veterinary antibiotic residue screening, supporting food safety surveillance efforts.

## 1. Introduction

Human health is closely affected by the environment, particularly the quality and safety of food. According to Acosta et al. [1], the global consumption of veterinary antibiotics in livestock and poultry farming was estimated to have reached 110,777 tons in 2019 and is projected to increase to 143,481 tons by 2040. This reflects the rising demand for animal-based food products and the need for effective control of infectious diseases in livestock populations. To address these needs, the use of veterinary antibiotics has been steadily increasing [2,3]. To mitigate the risk of antimicrobial resistance, many countries have restricted the use of veterinary antibiotics to therapeutic purposes only; for example, Europe and the USA have banned their use as growth promoters [4]. Veterinary antibiotics play a critical role in treating and preventing animal diseases, mitigating malnutrition, and regulating physiological, psychological, and biological functions to support animal health [5]. However, residues of these veterinary antibiotics in meat products pose considerable risks to human health. These risks include antimicrobial resistance [6], carcinogenic effects, acute toxicity, allergic reactions, and disruption of the intestinal microbiota [7,8,9,10].

Colloidal gold immunoassay (CGIA) is a rapid detection method based on antigen–antibody immune reactions and is widely used in veterinary antibiotic residue detection, food safety testing, and environmental monitoring [11,12,13,14]. Due to its fast detection speed, ease of operation, and minimal equipment requirements, CGIA has become the preferred method for on-site rapid screening. Antigen–antibody test cards, which are commonly employed in CGIA, are extensively utilized in medical diagnostics, animal disease prevention, food safety control, and environmental monitoring, owing to their portability, efficiency, and rapid response. For example, CGIA-based antigen–antibody test cards can be used to screen for residues of antibiotics such as sulfonamides, trimethoprim, and florfenicol in livestock. However, CGIA generally exhibits a relatively high detection limit, which restricts its suitability for high-precision detection scenarios [15]. Sun et al. [16] reported that the competitive indirect enzyme-linked immunosorbent assay (CI-ELISA) achieved a limit of detection (LOD) of 0.35 ng/mL for ofloxacin, while the corresponding CGIA method, showed a higher LOD of 10 ng/mL. Moreover, the interpretation of conventional antigen–antibody test cards primarily relies on visual assessment, which is prone to subjectivity and susceptible to variations in ambient lighting conditions, leading to inconsistent results and compromised reliability. However, image-based methods can keep the environment and lighting consistent. With trained models, they also reduce subjectivity in visual interpretation. For example, they can minimize differences among individuals, including those with color weakness.

In recent years, researchers worldwide have actively explored the application of image processing and artificial intelligence technologies in the interpretation of diagnostic test cards, aiming to enhance detection accuracy and efficiency. To address the challenge of missed detections caused by the difficulty of visually identifying weakly positive results, Miikki et al. [17] constructed a windowed macro-imaging system based on Raspberry Pi to collect a dataset, and analyzed the RGB images of the test regions to interpret results. However, such RGB image-based analytical approaches often fail to account for the complexity of real-world conditions such as variable lighting and camera angles, and as a result, the system’s robustness is limited. In the frameworks described in [18,19], users are required to capture test card images using a smartphone or tablet and upload them to a remote server for processing. While this architecture supports centralized computation, it suffers from significant limitations in scenarios with unstable network connectivity, potentially leading to delays or failures in result delivery and thereby compromising real-time performance and reliability. Turbé et al. [20] categorized the annotated images into two classes based on the type of HIV rapid diagnostic test (RDT), and compared the performance of support vector machines (SVMs) [21] and convolutional neural networks (CNNs). Their study identified MobileNetV2 as the most suitable model, demonstrating that deep learning can substantially improve the reliability and generalizability of on-site rapid testing. Lee et al. [22] proposed a deep learning-assisted diagnostic system named TIMESAVER. Unlike conventional binary classification methods, this study categorized test results into five classes based on real-world data, enabling more comprehensive analysis of false positives and false negatives. To achieve precise classification, Dastagir et al. [23] employed YOLOv8 to detect and localize the membrane region of the test card, which serves as the region of interest (ROI) for subsequent interpretation. The extracted ROI images were then converted to grayscale and resized before being input into a custom-designed CNN model. These studies demonstrate the value of deep learning and image processing for interpreting antigen–antibody test cards. They mainly focus on single-card analysis. As a result, they lack the capability for high-throughput interpretation, which limits their overall detection efficiency. In addition, due to the scarcity of standardized benchmark datasets for test cards, most existing studies have relied on bespoke collections.

To enable more scalable and automated test card interpretation, more and more researchers have begun turning to object detection algorithms. Object detection does not involve classifying an entire image, but having the computer both find and identify the region of interest (result discrimination area) within an image that might contain multiple cards.These algorithms can automatically locate and classify the test regions within an image. This capability is crucial for high-throughput analysis, where many cards need to be processed quickly and consistently. Currently, object detection algorithms can be broadly categorized into two types: two-stage and one-stage approaches. Two-stage algorithms generate possible regions of interest and then analyze each region. This is similar to a clinician identifying suspicious areas on an X-ray and then examining them in detail. While this process usually achieves higher accuracy, it requires more computing time. Representative models in this category include the Region-based Convolutional Neural Network (R-CNN) series, which includes R-CNN [24], Fast R-CNN [25], and Faster R-CNN [26]. R-CNN generates many possible regions in the image, and then classifies each region separately. This method is accurate, but very time-consuming. Fast R-CNN improves efficiency by extracting features from the image and classifying candidate boxes within the same network. Faster R-CNN further enhances the process by allowing the computer to automatically suggest the most likely regions, instead of checking every possible area. As a result, the algorithm becomes much faster while still maintaining high accuracy. In contrast, one-stage algorithms analyze the entire image in a single step, directly predicting the location and category of objects. This design is simpler and much faster, which makes it particularly suitable for real-time or embedded applications. Representative examples include Single-Shot MultiBox Detector (SSD) [27] and the You Only Look Once (YOLO) series [28,29,30,31,32,33,34,35,36]. Notably, YOLOv9 introduces several improvements that make it faster and more efficient: it can better capture both large and small details in an image, while keeping the model size and computing requirements relatively low. Building on this, YOLOv10 further refines the network structure and training methods, enhancing both accuracy and speed. Xie et al. [37] proposed YOLO-ACE, an improved YOLOv10 model for vehicle and pedestrian detection in autonomous driving scenarios. They introduced a novel double distillation strategy to transfer knowledge from a stronger teacher model to a student model. In addition, they redesigned the network structure to improve both detection speed and accuracy.

To overcome the limitations of manual interpretation and CGIA’s inherent shortcomings, we developed VetStar—a portable, cost-effective system for veterinary antibiotic residue detection for primary food safety laboratories. These laboratories, which are often located in remote areas with unstable network connectivity, require devices capable of offline, high-throughput analysis. Therefore, the system must support real-time image acquisition, result interpretation, and reporting without reliance on cloud-based services.The core challenge of this study is to efficiently perform object detection of antigen–antibody test cards on resource-constrained embedded devices. Because of the real-time demands of on-site processing, a high detection speed is essential. While the YOLO series of algorithms have achieved a good balance between speed and accuracy, our tests using YOLOv8n, YOLOv9t, YOLOv10n, and YOLOv11n to detect antigen–antibody test card images on the RK3568 platform showed that the average inference time exceeded 20 s, which exceeds the acceptable limits for practical use.

Lightweight deep learning models could potentially improve the inference speed without significantly sacrificing the accuracy. For example, Ma et al. [38] demonstrated that the Star Operation can map input to a high-dimensional nonlinear feature space and proposed the StarNet structure as a compact and effective deep learning network. Ding et al. [39] introduced the RepVGG architecture, which employs different topological structures during the training and inference stages. Through reparameterization, it simplifies into a single-branch structure at the inference stage, thus improving inference speed. Additionally, Yang et al. [40] proposed a logic-based knowledge distillation method: Bridging Cross-task Protocol Inconsistency Knowledge Distillation (BCKD), a machine learning technique distinct from traditional chemical distillation. This method enhances distillation by mapping classification logits into multiple binary classification tasks, allowing the student model to achieve higher accuracy without increasing its parameter count. Building on these prior advancements, we designed a small, lightweight, and efficient object detection model tailored for interpreting antigen–antibody test cards.

The main contributions of this paper are as follows:

1. We develop a portable, AI-powered veterinary antibiotic residue detection system that enables rapid, high-throughput interpretation of antigen–antibody test cards, and is designed for both laboratory and field use to improve efficiency and reduce subjectivity.
2. We present VetStar, a lightweight and accurate detection model tailored for embedded devices. It incorporates StarBlock for efficient feature extraction and a novel Depthwise Separable–Reparameterization Detection Head (DR-head), keeping accuracy while reducing parameters compared to YOLOv8 and YOLOv11.
3. We apply BCKD to train VetStar, enabling it to distill knowledge from a larger teacher model without an increase in the parameter count, enhancing accuracy while maintaining compactness.

## 2. Results and Discussion

### 2.1. Evaluation Metrics

In object detection tasks, the performance of neural networks is typically assessed using key metrics such as precision, recall, mean average precision (mAP), parameters, floating-point operations per second (FLOPs), and inference time (Latency). These metrics collectively provide a comprehensive evaluation of both model accuracy and computational efficiency. Based on the relationship between the predicted bounding box and the ground truth, prediction outcomes can be categorized into four types: true positive (TP), false positive (FP), false negative (FN), and true negative (TN). Specifically, TP represents correctly detected objects, FP denotes incorrect detections, FN refers to missed or misclassified objects, and TN corresponds to background or negative-class predictions. In this study, the antigen–antibody test card classification task involves only two categories: negative and positive. However, within the object detection framework, the predicted bounding boxes are classified into three categories: negative, positive, and background.

In this experiment, class-wise precision and class-wise recall were calculated for each class. The overall precision and recall were then obtained using the macro-average method, which computes the arithmetic mean across all classes. For all evaluations, both the Intersection-over-Union (IoU) threshold and the confidence threshold were set to 0.5, without additional confidence calibration. Here, IoU measures the overlap between the predicted bounding box and the ground truth. Confidence indicates the model’s certainty about the predicted class of an object within a bounding box.

(1) $$Precision=\frac{TP}{TP+FP}$$

(2) $$Recall=\frac{TP}{TP+FN}$$

Precision measures the proportion of samples predicted by the model as positive that are actually positive, thus reflecting the model’s prediction accuracy. Recall, on the other hand, measures the proportion of actual positive samples that are correctly predicted by the model, indicating the model’s ability to cover all positive samples. Average Precision (AP) is a core metric used to evaluate the performance of an object detection model on a single category. It reflects the combined effect of precision and recall. Mean Average Precision (mAP) is obtained by taking the arithmetic mean of AP values across all categories. It is the most widely used metric in object detection and represents the overall performance of the model. mAP50 refers to the mean AP when the IoU threshold is fixed at 0.5. mAP50-95 is calculated as the average AP over ten IoU thresholds, ranging from 0.5 to 0.95 in increments of 0.05. This provides a more comprehensive evaluation under different levels of strictness. Our dataset contains no overlapping objects, and highly precise localization is not required. Therefore, this study places particular emphasis on using mAP50 to evaluate model performance. The formula for calculating mAP is as follows:

(3) $$\mathrm{mAP}=\frac{1}{N}\sum_{i=1}^{N}{\mathrm{AP}}_{i}$$

where N represents the number of categories and ${AP}_{i}$ represents the average accuracy of category i.

Indicators such as Parameters, FLOPs, and Latency are generally used to measure the efficiency, computing resource requirements, and real-time performance of the model, respectively. Parameters are the sum of the parameters across all layers of the network. As shown in (4), *L* denotes the total number of layers in the network and ${Params}_{l}$ represents the number of parameters in the *l*-th layer. The term FLOPs refers to the number of floating-point operations required by the model in a forward propagation. In (5), $C_{l}^{in}$ represents the number of input channels of the *l*th layer, $C_{l}^{out}$ represents the number of output channels of the *l*th layer, $K_{l}$ is the size of the convolution kernel of the *l*th layer, $H_{l}$ and $W_{l}$ represent the height and width of the output feature map of the *l*th layer, respectively. Latency is the time required for the model to process a single sample. The formula is as follows:

(4) $$\mathrm{Parameters}=\sum_{l=1}^{L}{\mathrm{Params}}_{l}$$

(5) $$\mathrm{FLOPs}=\sum_{l=1}^{L}\left(2\cdot C_{l}^{\mathrm{in}}\cdot C_{l}^{\mathrm{out}}\cdot K_{l}^{2}\cdot H_{l}\cdot W_{l}\right)$$

(6) $$\mathrm{Latency}=t_{\mathrm{end}}-t_{\mathrm{start}}$$

### 2.2. Comparison Experiments on Bottom Plate Color

**Figure 1:**
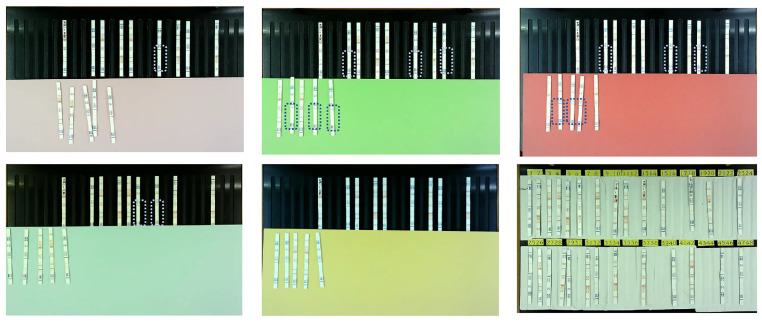
Comparative experiment on different bottom plate colors. The framed test strips indicate that the coloration regions are difficult to distinguish due to the plate color.

When constructing the hardware module for the veterinary antibiotic residue detection system, we initially used black bakelite material for the bottom plate. However, we observed that photos of the test cards taken with the black bottom plate exhibited light reflections, creating a layer of white fog that made the colors of some test cards difficult to identify in the images. To address this issue, we designed a bottom plate color control experiment. As shown in Figure 1, we compared A4 papers of different colors. The framed area in the figure indicates the region where the color is not clearly visible. The experimental results revealed that black, green, and red bottom plates caused the test card’s color to be obscured, whereas the white bottom plate allowed the test card’s colors to be the clearest. Based on these findings, we ultimately selected white PVC material for the bottom plate of the hardware module.

### 2.3. VetStar Experiments and Comparison with YOLO Series

To evaluate the performance of the proposed VetStar, we trained each model with three different random seeds (0, 1, and 10). The results are reported as the mean and standard deviation across these three runs. We compared it with YOLOv8n, YOLOv9t, YOLOv10n, and YOLOv11n on the test set of the VDR-RTC dataset. As shown in Table 1, VetStar (post-reparameterization) has significantly fewer Parameters and FLOPs than the smallest model in the YOLOv8-v11 series. Specifically, VetStar has around 1/67 of the Parameters of YOLOv10n and 1/27 of the FLOPs of YOLOv10n, highlighting its great suitability for deployment on an embedded system.

In terms of detection accuracy, VetStar achieves an average mAP50 of 96.1% across the three random seeds. This value is only 1.6 percentage points lower than that of YOLOv11n, the best-performing model under the mAP50 metric. For mAP50-95, VetStar is lower than 3.2% behind YOLOv8n, which shows that it is less effective for high-precision localization. However, our task does not require such fine-grained positioning. It only needs to detect the approximate card region to determine whether the result is positive or negative. Therefore, we primarily use mAP50 to evaluate the detection performance of our model.

Overall, these results show that the VetStar model, which was built using the lightweight StarBlock module and DR-head, not only offers substantial reductions in model size, but also maintains strong feature representation and competitive detection performance.

### 2.4. Comparison Experiments on Detection Head

Our DR-head is an enhanced design based on the YOLOv8-head. We introduce a shared layer composed of DW-Conv and RepConv. While RepConv employs a multi-branch structure during training to improve feature extraction, it simplifies to a single-branch structure during inference. This design achieves lightweight computation without sacrificing the model’s feature extraction capabilities. The experimental results are shown in Table 2. When integrated into VetStar, the  DR-head has only 0.04 M Parameters and 0.3 G FLOPs, which are 0.06 M and 0.2 G lower than the detection heads in YOLOv8 and YOLOv11, respectively. Despite this reduction, Precision, Recall, and mAP50 all show significant improvements. Specifically the mAP50 of DR-head is 2.6 percentage points higher than that of the YOLOv8-head.

**Figure 2:**
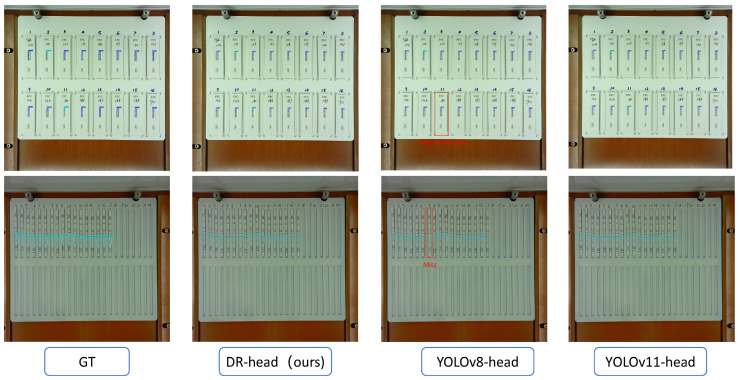
Detection results of VetStar under different detection heads. Dark blue detection boxes indicate positive detections, light blue boxes indicate negative, and red boxes highlight classification errors or missed detections.

**Figure 3:**
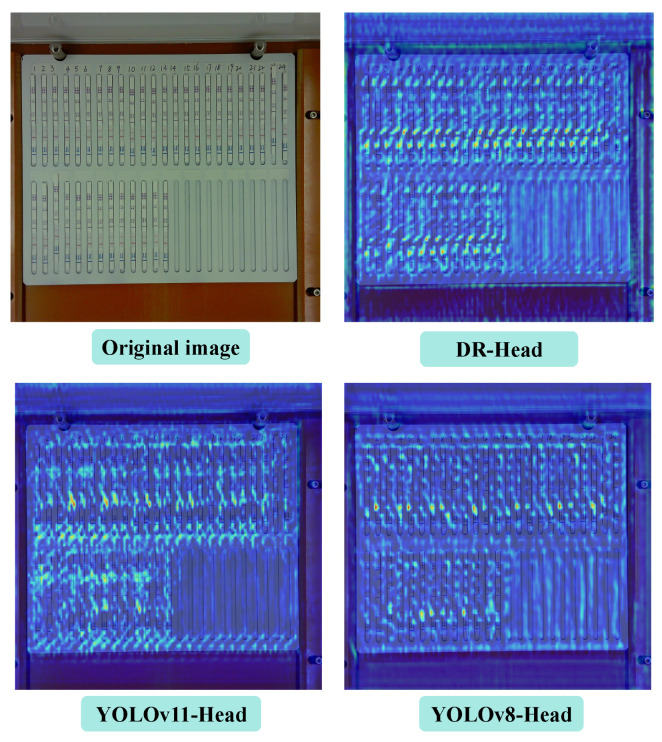
Comparison of heatmap produced by different detection heads.

Figure 2 displays the detection results of VetStar using different detection heads. YOLOv8-head suffers from both misclassifications and missing detection boxes. Figure 3 presents heatmap comparisons of VetStar with different detection heads. The heatmaps show that VetStar with the DR-head focuses more effectively on the color development region of the test strip, which reduces missed detections and improves detection accuracy. These findings show that the proposed DR-head has strong recognition performance in the task of antigen–antibody test card interpretation.

### 2.5. Knowledge Distillation of VetStar Using BCKD

In all distillation experiments, the ratio of distillation localization loss to classification loss was set to 15:1 ($\alpha_{1}:\alpha_{2}$ in (19)). The average epoch training time was 11.2 s. The teacher model was YOLOv11n-one head, trained for 359 epochs with only the C4-level detection head retained. Table 3 shows the results of hyperparameter tuning for λ in the knowledge distillation process. λ represents the proportion of distillation loss in the total loss function used to train VetStar. As can be seen from the table, we varied λ from 1.0 to 1.5, increasing it by 0.1 at each step, and performed model validation after each adjustment. The experimental results show that when λ is adjusted from 1.0 to 1.3, mAP50 continues to increase, and when λ=1.3, Precision, Recall, and mAP50 all reached their highest values. Therefore, λ=1.3 was used in all subsequent experiments.

We employed the BCKD method to train VetStar. No additional class imbalance remedies, such as focal loss or hard-negative mining, were applied during training. BCKD is a logic-based knowledge distillation method, and and models rely on detection heads to generate judgment criteria and scores. BCKD requires that the number of detection heads in the student model match those in the teacher model during the distillation process. Therefore, we selected the YOLOv11n model, which has a single detection head at the C4 level, as the teacher model, while VetStar served as the student model. The experimental results are shown in Table 4. We compared Distilled-VetStar with YOLOv8n, YOLOv9t, YOLOv10n, YOLOv11n, YOLOv11(one head), and the original VetStar before distillation. After applying knowledge distillation using the YOLOv11-one-head teacher model, VetStar’s mAP50 increased by 1.2 percentage points, reaching the same accuracy level as YOLOv11-one-head, with the Recall improved by 1 percentage point. This indicates that under the guidance of YOLOv11-one-head, VetStar successfully learned subtle differences between the positive and negative features, even identifying negative samples with faint visual cues. The final trained Distilled-VetStar achieved the second-highest mAP50, which was only 0.4% lower than the best YOLOv11n, with Precision and Recall results differing by less than one percentage point compared to other models. Furthermore, the Parameters and FLOPs for Distilled-VetStar were significantly reduced, providing an optimal balance between accuracy and computational power, making it well-suited for interpreting antigen–antibody test card results on embedded systems.

### 2.6. Performance Comparison on Embedded Devices

**Figure 4:**
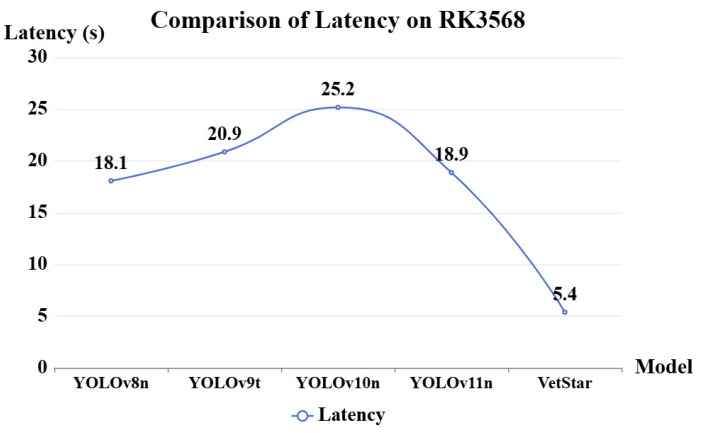
Comparison of inference Latency on the RK3568 embedded device.

The experiment in the previous section demonstrates that the VetStar algorithm, after knowledge distillation, achieves comparable accuracy to the YOLO series (v8-v11) in antigen–antibody test card results. In this section, we deploy the model on the RK3568 embedded device and compare the average Latency per image on the same hardware. The evaluation was conducted with a batch size of 1, after warm-up, under single-threaded execution. The model weights were converted from a PyTorch.pt file to ONNX format and inference was performed using ONNX Runtime, without additional quantization or NPU/DLA acceleration. The reported latency reflects pure model inference time and does not include image input or report generation. As shown in Figure 4, VetStar achieves an average latency of 5.4 s per image, which is 4.6 times faster than the slowest model, YOLOv10n, and 13.5 s faster than YOLOv11n, which, despite having the highest mAP50, exhibits the slowest inference speed. These results indicate that VetStar is better suited for high-throughput antigen–antibody test card result interpretation on embedded systems compared to the YOLO series models. Despite achieving comparable detection accuracy, VetStar offers significantly faster inference speed and greater practical application value.

### 2.7. Discussion on Diagnostic Metrics

Medical and veterinary diagnostic studies are commonly evaluated using accuracy, sensitivity, specificity, Positive Predictive Value (PPV), and Negative Predictive Value (NPV). Their definitions are given by the following formulas.

(7) $$Accuracy=\frac{TP+TN}{TP+TN+FP+FN}$$

(8) $$Sensitivity=\frac{TP}{TP+FN}$$

(9) $$Specificity=\frac{TN}{TN+FP}$$

(10) $$PPV=\frac{TP}{TP+FP}$$

(11) $$NPV=\frac{TN}{TN+FN}$$

However, sensitivity, specificity, PPV, and NPV are defined for binary classification, where samples only belong to two categories: positive and negative. In object detection, the model must not only classify but also localize objects, and each region in an image can be classed as positive, negative, or background. In this multi-class setting, sensitivity, specificity, PPV, and NPV are not strictly applicable. The reason is that, from the perspective of each class, that class is considered positive and all other classes are negative, which makes the definition of true negatives ambiguous. Nevertheless, analogous metrics exist in the multi-class case. As shown in (2) and (8), the definition of sensitivity is the same as recall. Similarly, as shown in (1) and (10), the definition of PPV is the same as precision. Sensitivity can therefore be interpreted as the recall of the positive class, and PPV as the precision of the positive class. By symmetry, if the negative class is viewed as positive, then the recall of the negative class can be interpreted as specificity, and the precision of the negative class as NPV. As stated in the previous experiments, the reported precision and recall were calculated using the macro-average method, which takes the mean of positive-class and negative-class precision and recall. Thus, the overall recall can be interpreted as a combined assessment of sensitivity and specificity, while the overall precision can be interpreted as a combined assessment of PPV and NPV. Moreover, mAP integrates both precision and recall, and is therefore the most representative metric for evaluating overall performance. As presented in Table 5, after knowledge distillation, VetStar achieved a 2.2% increase in positive recall. This indicates that by learning from YOLOv11, VetStar reduced missed and incorrect detections of positive cases. For the other metrics, VetStar and the compared models performed similarly, with differences within 1%.

**Figure 5:**
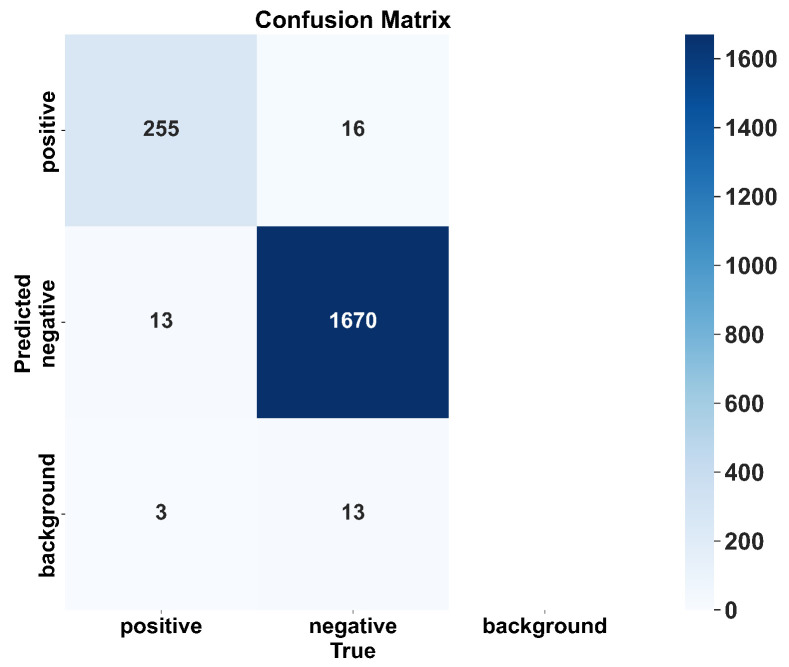
Confusion matrix of distilled-VetStar on the test set.

Because true negatives cannot be strictly defined, ROC analysis is not applicable in this context. Instead, we provide the confusion matrix of the distilled-VetStar on the test set in Figure 5. The test set contained 129 images with 1970 annotated objects, including 271 positive and 1699 negative ground-truth boxes. The confusion matrix shows that the distilled-VetStar correctly recognized most positive and negative classes. However, some positives and negatives were still misclassified or missed (i.e., interpreted as background).

## 3. Materials and Methods

### 3.1. Veterinary Antibiotic Residue Detection System

#### 3.1.1. System Architecture

**Figure 6:**
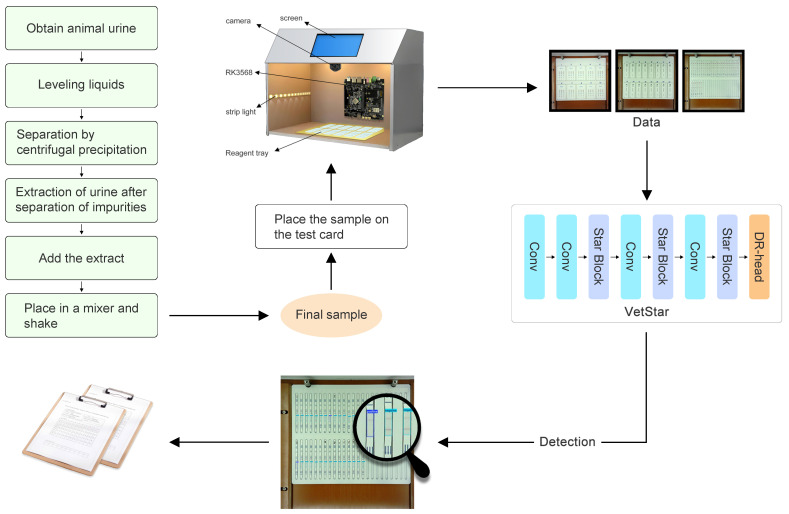
Flowchart of the veterinary antibiotic residue detection system for food safety laboratories.

As illustrated in Figure 6, the sample shown is prepared from pig urine, although samples can also be prepared from pig meat, chicken meat, duck meat, and other animal-derived materials. After collecting animal urine, the experimenter performs a colloidal gold assay and dispenses the processed sample onto a veterinary antibiotic residue test card. Once the color development is complete, the card is then placed into the designated tray of the veterinary antibiotic residue detection system. The system’s hardware captures an image of the test card, and the built-in RK3568 embedded chip performs model inference to analyze the test results. The inference output is then automatically compiled into an experimental report, which is saved in PDF format. Furthermore, the system supports both wired and wireless network connectivity to transmit experimental data to the veterinary antibiotics supervision platform via an Application Programming Interface (API). This functionality not only enhances the efficiency of veterinary antibiotic residue detection workflows but also facilitates real-time data sharing, thereby improving inter-laboratory collaboration and regulatory traceability.

#### 3.1.2. Hardware Module

The interpretation of colloidal gold antigen–antibody test cards relies on the chromogenic region of the test line (T line) and the control line (C line), considering both the presence and intensity of color. We adopt the line elimination method to determine the results of the test cards (see Table 6). However, manual interpretation of colloidal gold antigen–antibody test cards has two main limitations. First, individuals’ sensitivity to color variations differs, which may lead to inconsistencies when it comes to judging the T line color. Second, ambient light intensity significantly affects color perception, and inadequate lighting conditions can further amplify interpretation errors. To address these challenges, we designed and implemented an integrated testing device featuring an embedded system and a built-in camera. This device captures test card images using the camera and performs high-throughput detection and analysis on the embedded platform, ensuring efficient and reliable test results. Additionally, the system synchronously displays the test card’s location and interpretation results on the client UI (user interface, the interactive interface between the user and the software), allowing users to intuitively view and manage experimental data.

**Figure 7:**
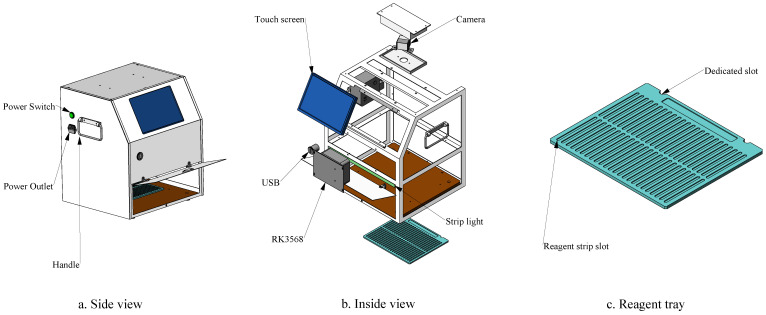
Prototype diagram of the hardware module.

As shown in Figure 7a, the hardware module of the veterinary antibiotic residue detection system features a compact box structure with a portable handle, making it convenient for both laboratory and field use while fully meeting portability requirements. The device is equipped with an embedded 10.1-inch high-definition touchscreen, enabling real-time test result display and acting as an intuitive operation interface, greatly enhancing user interaction. As depicted in the internal view (Figure 7b), a five-megapixel OV5640 autofocus USB camera (60° field of view) is integrated at the top to accurately capture the chromogenic region of the test card. The camera was positioned at a working distance of 40 cm, ensuring clear and high-resolution image acquisition for analysis. The device houses an RK3568 chip to run the VetStar object detection algorithm, enabling fast and efficient automated test card interpretation. To enhance stability and accuracy, the device uses a fixed COB LED strip light source (6000 K, 5 W/m) to provide uniform illumination and reduce external lighting interference. This light source produces minimal heat and does not cause photobleaching of the samples. A dedicated slot at the bottom allows users to easily insert the sample tray (Figure 7c). The tray supports three different specifications, making it compatible with various test strips and test cards, thereby meeting high-throughput detection needs. For connectivity, the device includes both network cable and USB interfaces and also supports WiFi, allowing it to be seamlessly integrated into networked environments when available. With cost-effectiveness in mind, the design ensures affordability for small laboratories by striking a balance between simplicity, practicality, and functionality to meet the needs of resource-constrained environments while efficiently supporting embedded system applications.

#### 3.1.3. Software Modules

**Figure 8:**
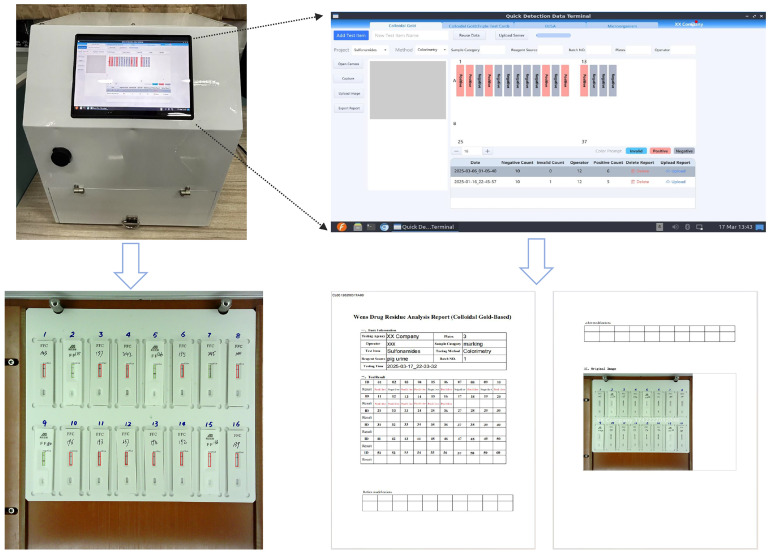
Graphical user interface and report export functionality of the detection system.

To enhance user convenience and interactive experience, we have developed a client-side software for the veterinary antibiotic residue detection system. As shown in Figure 8, after completing the colloidal gold assay, the experimenter applies the sample onto the colloidal gold antigen–antibody test card and places it on the tray. The tray is then inserted into the system’s hardware module, which automatically captures an image of the test card. Through the client software, the experimenter can capture the reagent tray image with a single click and invoke the VetStar model to determine whether the results are positive or negative. The system then generates a structured JSON file containing the test results. The JSON file contains the reagent source, the testing company, the positive or negative results in location order, and other related information. It does not include image hashes (see Appendix A). Users can directly export the report as a PDF and upload it to the remote veterinary antibiotics supervision platform via the software, ensuring real-time data sharing and standardized management of experimental records.

### 3.2. Methods

In this section, we begin by introducing the construction process of our dataset. Then, considering real-world application scenarios, we integrate the efficient feature extraction module StarBlock into VetStar, forming the backbone network. Based on this foundation, we propose the DR-head structure to effectively reduce the size of the model. To further enhance VetStar’s detection accuracy, we adopt a logic-based knowledge distillation method, using the single-detection-head YOLOv11n as the teacher model to guide VetStar’s training. Through these optimizations, the proposed VetStar model achieves an optimal balance between detection accuracy and inference efficiency on our self-constructed dataset.

#### 3.2.1. Data Acquisition

All the data in this study were collected from pig farms in Xinxing County, Yunfu City, Guangdong Province, China. Urine samples were obtained from pigs and then applied to colloidal gold-based veterinary antibiotic residue test strips (cards) according to standard procedures. In total, 647 images of three different types of test strips (cards) were collected, with an original resolution of 2592 × 1944 pixels. Since some of the background information in the images is irrelevant to the detection task, we cropped the original images to a uniform resolution of 1944 × 1944, resulting in the construction of the Veterinary Drug Residue Rapid Test Card (VDR-RTC) dataset.The VDR-RTC dataset contains three types of test strip (card) formats:

1. Single-strip images—each image contains up to 48 test strips.
2. Single-card images—each image contains up to 16 test cards.
3. Triple-card images—each image contains up to 10 test cards, with each card detecting residues of three different drugs simultaneously.

The data annotation only marked the color development region, rather than the entirety of each test card. Annotation was performed by three individuals using Labelme. In cases of disagreement, the label agreed upon by the majority was adopted. The annotation procedure for triple-card images was the same as that for single-strip and single-card images, with all annotations focusing on the color development region. Since the invalid class is extremely rare and the model cannot effectively learn its characteristics, the VDR-RTC dataset does not include test cards with invalid results.

**Figure 9:**
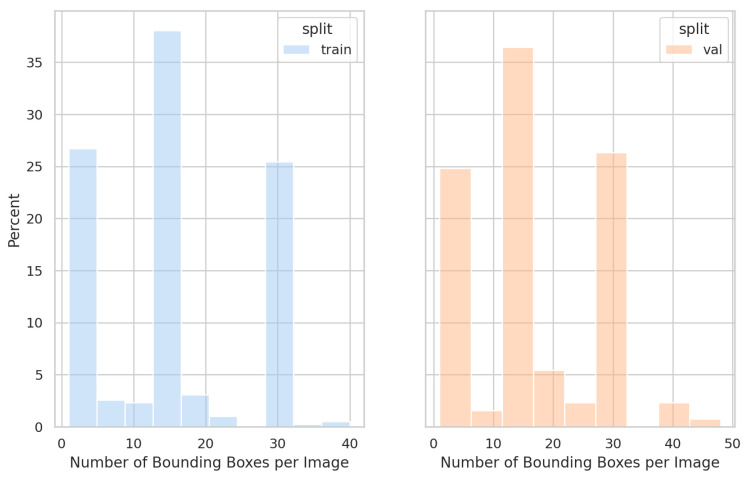
Distribution of bounding box counts per image in the dataset.

The VDR-RTC dataset includes a total of 10,225 annotated bounding boxes, consisting of 8486 negative boxes and 1736 positive boxes. It was divided into training, validation, and test sets in a ratio of 6:2:2, with all three types of test cards evenly distributed across the subsets. The training set contains 389 images with 6026 bounding boxes (4983 negative and 1043 positive). The validation set contains 129 images with 2229 bounding boxes (1804 negative and 425 positive). The test set contains 129 images with 1970 bounding boxes (1699 negative and 271 positive). Figure 9 presents the distribution of bounding boxes per image across the VDR-RTC training and validation sets.

#### 3.2.2. Construction of Vetstar Model

**Figure 10:**
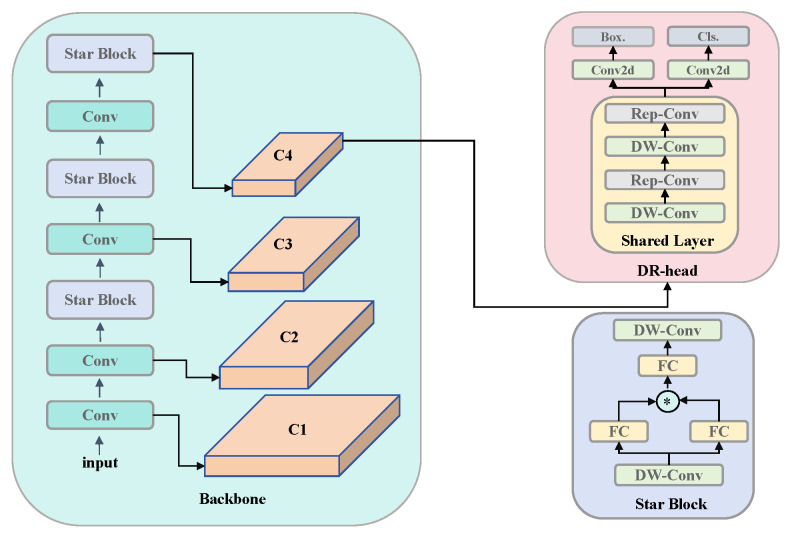
Overview of the proposed VetStar architecture. Conv: convolution; Conv2d: two-dimensional convolution; DW-Conv: depthwise convolution; Rep-Conv: re-parameterized convolution; FC: fully connected layer; Cls: classification; Box.: bounding box regression; C1–C4: multi-scale feature maps extracted from successive backbone stages.

Our lightweight VetStar model consists of StarBlock, convolutional layers (Conv), and DR-Head, as illustrated in Figure 10. The specific structure of DR-Head is detailed in the next subsection. StarBlock is primarily composed of Depthwise-separable convolution (DW-Conv) and fully connected layers (FCs). The initial DW-Conv layer efficiently extracts spatial information from the feature maps. This is followed by a dual-branch FC that performs linear mapping. Feature transformation is enhanced through element-wise multiplication. This is referred to as the Star Operation, projecting the features into a high-dimensional space to improve representation capability. Another FC further fuses the high-dimensional features, and a second DW-Conv is then applied to reintegrate spatial information, ensuring that the output features retain both rich semantic details and sufficient spatial structure.

The lightweight and efficient nature of StarBlock stems from its ability to implicitly map features into a high-dimensional nonlinear space through Star Operation without explicitly increasing the network’s width and depth. Given an input feature $f\in R^{d}$, it is extended to $f^{\prime }\in R^{d+1}$ by adding a bias term. After two linear transformations, $M_{1}^{T}f^{\prime }$ and $M_{2}^{T}f^{\prime }$, the element-wise multiplication can be expressed as

(12) $$\left(M_{1}^{T}f^{\prime }\right)*\left(M_{2}^{T}f^{\prime }\right)=\sum_{i=1}^{d+1}\sum_{j=1}^{d+1}m_{1i}m_{2j}f_{i}^{\prime }f_{j}^{\prime }$$

This operation generates combinations of $f_{i}^{\prime }f_{j}^{\prime }$, resulting in approximately $\frac{\left(d+2)(d+1\right)}{2}\approx \frac{d^{2}}{2}$ independent terms. This means that although the original input feature dimension is only *d* (or d+1 after adding the bias term), after applying the Star Operation, the implicit feature space expands to roughly $\frac{d^{2}}{2}$ dimensions. When multiple StarBlocks are stacked, the feature dimensionality increases further. For instance, stacking two layers of StarBlock expands the feature space, and stacking ${\left(\frac{d^{2}}{2}\right)}^{2}$ n layers extends it to ${\left(\frac{d^{2}}{2}\right)}^{2^{n}}$. However, excessive expansion of feature dimensions may lead to redundancy, increasing the risk of overfitting. Based on our experimental results, we found that using a single layer of StarBlock at each level achieves an optimal balance between feature enhancement and computational efficiency.

VetStar is a compact network optimized for recognizing antigen–antibody test card results and is specifically designed for fixed-position detection tasks. In the veterinary antibiotic residue detection system, our primary objective is to identify antigen–antibody test cards within a predefined region, eliminating the need for multi-scale feature fusion. Based on experimental analysis, we found that VetStar’s feature maps at the C4 level adequately cover the effective detection region for all test cards. Therefore, to further reduce computational overhead and enhance inference efficiency, we connect the DR-Head exclusively to the C4-level feature maps to generate the corresponding prediction boxes.

#### 3.2.3. DR-Head

**Figure 11:**
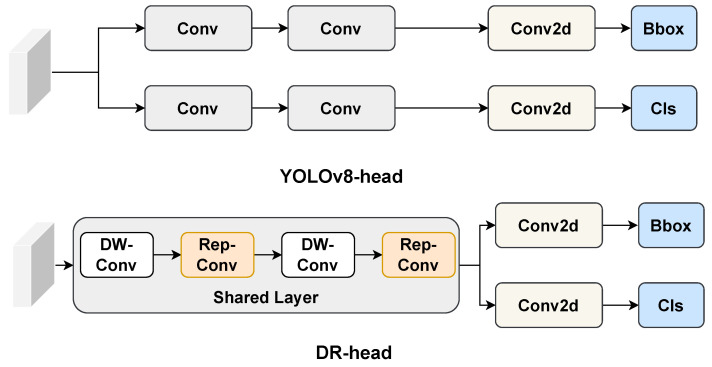
Structural comparison between the YOLOv8-head and the proposed DR-head. Conv: convolution; Conv2d: two-dimensional convolution; DW-Conv: depthwise convolution; Rep-Conv: reparameterized convolution; Bbox: bounding box regression; Cls: classification.

In Figure 11, the upper section illustrates the structure of the YOLOv8 detection head, while the lower one presents the VetStar detection head. In YOLOv8, the detection head accounts for nearly half of the model’s total parameters. It uses a decoupled head design, separating classification and regression into two independent branches. This approach effectively minimizes interference between the two tasks, enhancing detection accuracy in complex scenes and multi-category detection scenarios. However, compared to a coupled head, the decoupled design significantly increases computational complexity and parameter count. To address this, YOLOv11 introduces DW-Conv in the classification branch, reducing both computational cost and parameter count by decoupling spatial and channel convolutions.

In antigen–antibody test card recognition, the target categories are limited to negative, positive, and background, and the background is relatively simple. Leveraging this characteristic, we developed DR-Head based on the YOLOv8 detection head, adopting a partially coupled structure: the initial layers use shared convolution, while the latter layers are divided into separate classification and regression branches. This design allows feature sharing between the two tasks, reducing computational complexity while mitigating the interference issues associated with a fully coupled structure.

In the shared convolution module, DW-Conv (input/output channels = 32, kernel size = 3 × 3, parameters = 352) and Reparameterization Convolution (Rep-Conv, input/output channels = 32, kernel sizes = 3 × 3 and 1 × 1, parameters = 10,368) are used in an alternating manner. DW-Conv efficiently extracts features with minimal computation, while Rep-Conv employs a multi-branch structure to enhance feature extraction capacity, which is merged into a single convolutional layer during inference to eliminate redundant computational overhead. This “train-complex, infer-efficient” strategy ensures high inference efficiency without compromising detection accuracy. Additionally, by integrating DW-Conv’s lightweight feature extraction with Rep-Conv’s parameter-intensive feature fusion, DR-Head effectively learns multi-scale features, improving robustness to object scale variations and deformations.

#### 3.2.4. BCKD

Although the VetStar model significantly outperforms the YOLO series (e.g., YOLOv8-v11) in real-time performance, inference speed, and resource efficiency, there remains a gap in recognition accuracy. In the high-throughput antigen–antibody test card recognition task, the test card’s target area occupies only a small portion of the entire image. Due to VetStar’s compact model size and the limited area requiring recognition, its ability to extract comprehensive feature information is constrained, leading to a decline in classification performance. This issue is particularly pronounced when sample impurities are present, as the secondary line in negative results may appear faint, increasing the likelihood of misclassification.

To address this challenge, we introduced a logic-based knowledge distillation method—BCKD. Knowledge distillation is a machine learning technique where a small “student model” learns from the experience of a large “teacher model”, enabling the small model to improve performance while remaining compact. It is categorized into two types: logic-based distillation, where the student learns the teacher’s decision logic such as judgment criteria and scores; and feature-based distillation, where the student learns the intermediate features extracted by the teacher during data processing such as line and color information. In this study, we adopted logic-based knowledge distillation, the BCKD method, allowing VetStar to learn the judgment logic of YOLOv11n in order to accurately identify subtle feature differences in test cards.

Traditional knowledge distillation in object detection aims to transfer the teacher’s experience to the student model. A critical challenge is that the scoring protocols for the predicted bounding box classes are not consistent between the teacher and the student models. Conventional distillation adopts an “overall comparison” strategy using the Softmax protocol. This approach smooths out differences and prevents the student from learning essential details. This inconsistency in evaluation rules is exactly what BCKD addresses. The teacher model uses a “score-by-score” strategy with the Sigmoid protocol, which preserves subtle differences, such as the distinction between blurred and clear lines on detection cards. Employing the Softmax protocol adjusts the class scores of the predicted bounding boxes so that the sum of all categories equals one. This process ignores the absolute value of each class score and removes the subtle differences between samples. When the scores of the teacher and student models satisfy a certain proportional relationship, the distillation loss under Softmax becomes zero. In this situation, the student model assumes that it has already learned enough and stops acquiring more detailed knowledge from the teacher. In contrast, the Sigmoid protocol (13) and (14) does not impose this summation constraint. It retains the absolute score of each category, so the differences between the teacher and student models remain visible after transformation. For example, if the teacher’s logits for a box are [background: 1.0, negative: 3.0, positive: 6.0] and the student’s are [1.0, 2.0, 4.0], Softmax normalizes both to nearly [0, 0, 1], making their outputs nearly identical and halting further learning. In contrast, using Sigmoid, the outputs would be [0.73, 0.95, 1.00] for the teacher and [0.73, 0.88, 0.98] for the student, clearly preserving the confidence gap for the positive sample(non-background category). These differences encourage the student model to continue learning until it can reproduce the teacher’s ability to recognize fine details, such as faint lines on test cards. In this way, the student does not stop learning too early, unlike in the Softmax case. The following equations define the Sigmoid transformation for both models, where $s^{s}$ represents the logits score of the student model, and $s^{t}$ represents the logits score of the teacher model.

(13) $$Sigmoid\left(s^{s}\right)=\frac{1}{1+e^{-s^{s}}}$$

(14) $$Sigmoid\left(s^{t}\right)=\frac{1}{1+e^{-s^{t}}}$$

**Figure 12:**
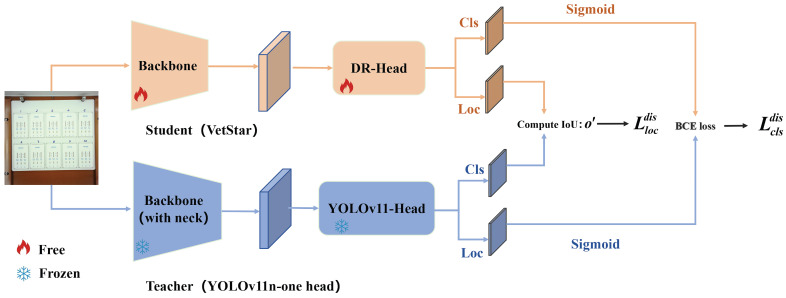
BCKD training pipeline applied to the VetStar model. The arrows indicate the direction of data flow; the parameters of the modules marked as ‘frozen’ are not updated during training. YOLOv11-Head: YOLOv11 with only one detection head; IoU: Intersection over Union; Cls: Classification; Loc: Localization; BCE loss: Binary Cross-Entropy loss.

BCKD computes the binary classification scores $s^{s^{\prime }}$ and $s^{t^{\prime }}$ of size N×K after applying the Sigmoid protocol. It treats the classification logits map as multiple binary classification maps and employs binary cross-entropy (BCE) loss to calculate the classification distillation loss. The BCE loss guides the student model to match the teacher’s judgment on each possible class independently, rather than averaging across all classes. This makes the learning more sensitive to subtle differences, such as faint lines on test cards. Specifically, $s_{i,j}^{s^{\prime }}$ and $s_{i,j}^{t^{\prime }}$ represent the classification scores at the i-th position and for the j-th class in $s^{s^{\prime }}$ and $s^{t^{\prime }}$. This approach calculates the loss independently for each category, allowing for a more fine-grained distinction between different classes. BCKD also introduces a loss weighting strategy to compute the weight β based on the difference in classification scores between the teacher model and the student model. As illustrated in Figure 12, the teacher model is the pre-trained YOLOv11n detection head, and the student model is our VetStar. Knowledge from YOLOv11n is transferred to VetStar through the BCKD method. First, an input image of a veterinary antibiotic test card is passed through the backbone of both models to extract key features. YOLOv11n also includes a neck module, which helps to capture features of objects at different scales. The extracted features are then processed by the detection heads of both models to predict the bounding box coordinates. After that, the detection heads classify the predicted bounding boxes. During training, VetStar computes the positional deviation of its predicted bounding boxes from those of YOLOv11n using $L_{loc}^{dis}$. It also calculates the classification deviation of the predicted class scores for each grid cell using $L_{cls}^{dis}$. These deviations are then used to update the parameters of VetStar’s backbone and detection head. In this way, the bounding box predictions and classification results of VetStar gradually approach those of YOLOv11n.

(15) $$L_{BCE}\left(s_{i,j}^{s^{\prime }},s_{i,j}^{t^{\prime }}\right)=-\left(\left(1-s_{i,j}^{t^{\prime }}\right)*log\left(1-s_{i,j}^{s^{\prime }}\right)+s_{i,j}^{t^{\prime }}*log\left(s_{i,j}^{s^{\prime }}\right)\right)$$

(16) $$\beta =|s^{t^{\prime }}-s^{s^{\prime }}|$$

The loss function is used to measure the difference between the student and teacher models. By minimizing this loss, the student model gradually improves and approaches the performance of the teacher model. The classification loss is defined as follows, where N represents the number of samples and K denotes the number of categories. The total classification distillation loss is given by

(17) $$L_{cls}^{dis}\left(x\right)=\sum_{i=1}^{N}\sum_{j=1}^{K}\beta_{i,j}\;*\;L_{BCE}\left(s_{i,j}^{s^{\prime }},\;s_{i,j}^{t^{\prime }}\right)$$

When traditional logits-based distillation methods apply distillation training to discrete position probability prediction heads, they often require modifications to the network model architecture. BCKD directly calculates the IoU of the bounding boxes predicted by the teacher and student models, obtains the positioning predictions $o_{i}^{t}$ and $o_{i}^{s}$ from the teacher and student models, and calculates the IoU between them–$o_{i}^{\prime }$. The position loss is as follows:

(18) $$L_{loc}^{dis}\left(x\right)=\sum_{i=1}^{N}Max\left(\beta_{.,j}\right)*\left(1-o_{i}^{\prime }\right)$$

The total distillation loss is composed of $L_{cls}^{dis}\left(x\right)$ and $L_{loc}^{dis}\left(x\right)$, where $\alpha_{1}$ and $\alpha_{2}$ are hyperparameters.

(19) $$L_{total}^{dis}\left(x\right)=\alpha_{1}*L_{cls}^{dis}\left(x\right)+\alpha_{2}*L_{loc}^{dis}\left(x\right)$$

VetStar’s total loss $L_{total}\left(x\right)$ consists of the loss between the predicted box and the true value $L_{total}^{gt}\left(x\right)$ and the distillation loss $L_{total}^{dis}\left(x\right)$, and λ is the weight hyperparameter of the total distillation loss.

(20) $$L_{total}\left(x\right)=\lambda L_{total}^{dis}\left(x\right)+L_{total}^{gt}\left(x\right)$$

### 3.3. Experimental Implementation

All experiments in this study were conducted on a server equipped with an AMD Ryzen 9 7900X 12-core processor, an NVIDIA GeForce RTX 4090D GPU, and 64 GB of RAM. The operating system was Ubuntu 22.04.5 LTS, and the framework used was PyTorch 2.2.0 with CUDA 12.1. Mixed-precision training (AMP) was enabled. To preserve as much of the original feature information as possible, the input image size for all models was set to 1952 × 1952, closely matching the original resolution. The batch size was set to 8, and training was conducted for a maximum of 500 epochs, with the Early Stopping technique applied to prevent overfitting. The Adam optimizer was used for all training processes. To ensure the consistency and fairness, all experiments reported in this study were conducted under the same conditions.

## 4. Limitations

This study has several limitations. First, while the system provides rapid and automated interpretation, a direct comparison with manual interpretation results was not included in this study. Such an analysis could provide additional insights into the advantages and limitations of automated detection. Second, although our system has already been deployed in 17 laboratories, where it is mainly used for detecting antibiotic residues in pig urine and chicken meat, the experiments reported in this paper were conducted only on pig urine samples. Other livestock-derived products, such as milk, meat, and poultry, could also be used for further validation to demonstrate the robustness and generalizability of the system. Future research will extend the evaluation to these sample types. Third, the evaluation of bottom plate color was limited to qualitative visual analysis. Quantitative measurements such as luminance or signal-to-noise ratio, together with detailed lighting spectrum and color-rendering index characterization, will be included in future work. Finally, the cost-effectiveness of the proposed system was not compared with conventional methodologies. A systematic cost analysis in future work would help highlight the economic advantages of our device for large-scale use.

Furthermore, the data were collected under fixed lighting conditions, which resulted in a limited dataset. No external datasets were used to validate the proposed model. Additionally, due to the challenges in collecting invalid results, we did not include invalid class modeling in this study. Future research will focus on gathering data from diverse regions, incorporating invalid classes, and conducting robustness testing to evaluate the model’s generalization under various conditions.

## 5. Conclusions

In veterinary antibiotic residue detection, colloidal gold antigen–antibody tests are often manually interpreted. However, due to variability in personnel and lighting conditions, interpretation results can be inconsistent. To accelerate the automation process in food safety laboratories, we developed a veterinary antibiotic residue detection system that operates on an embedded platform. Traditional intelligent interpretation methods for antigen–antibody test cards typically treat the task as an image classification problem. However, in order to achieve high-throughput result interpretation, we employed an object detection approach and implemented an efficient antigen–antibody test card detection system on the RK3568 chip. To improve detection efficiency, we proposed the VetStar algorithm, which is designed specifically for rapid and accurate antigen–antibody test card on the embedded hardware. VetStar incorporates a custom feature extraction module, StarBlock, which is known for its strong representation capabilities even in shallow networks. Additionally, we developed a lightweight detection head based on the YOLOv8 architecture, tailored for the antigen–antibody test card interpretation task. Compared to the YOLO series models evaluated in this study, VetStar demonstrates significant advantages in terms of model size, although a slight gap in detection accuracy remains. To further improve accuracy, we incorporated the logic-based knowledge distillation method, BCKD, using the YOLOv11n model with a single detection head as the teacher. After distillation, VetStar’s mAP50 accuracy increased to 97.4%, narrowing the accuracy gap with YOLOv11n to just 0.4%. Deployment experimental on the RK3568 embedded device revealed that VetStar is 4.6 times faster than YOLOv10n and 13.5 s faster than YOLOv11n, which achieved the highest mAP50. In conclusion, the veterinary antibiotic residue detection system we developed delivers both high detection accuracy and fast inference speed, making it suitable for the high-throughput result interpretation demands of colloidal gold antigen–antibody test cards in food safety laboratories.

## Figures and Tables

**Table 1:** Performance comparison between VetStar and YOLO series models (v8–v11). The results are averaged over three random seeds (0, 1, 10). Bold indicates the best performance among the compared methods.

Model	Precision (%)	Recall (%)	mAP50 (%)	mAP50-95 (%)	Parameters (M)	FLOPs (G)
YOLOv8n	95.8 ± 2.6	96.9 ± 0.6	97.3 ± 0.6	**91.7 ± 1.3**	2.68	6.8
YOLOv9t	94.9 ± 1.8	96.0 ± 1.2	97.2 ± 0.6	90.6 ± 0.6	1.73	6.4
YOLOv10n	96.2 ± 1.1	96.6 ± 0.8	97.4 ± 0.3	91.5 ± 0.4	2.69	8.2
YOLOv11n	**96.9 ± 0.7**	**96.9 ± 0.3**	**97.7 ± 0.5**	91.6 ± 0.9	2.58	6.3
VetStar	96.4 ± 0.6	94.8 ± 1.0	96.1 ± 0.1	88.5 ± 0.2	**0.04**	**0.3**

**Table 2:** Comparison of DR-head with YOLOv8-head and YOLOv11-head on VetStar. The results were obtained using a fixed random seed of 0. Bold indicates the best performance among the compared methods.

Detection Head	Precision (%)	Recall (%)	mAP50 (%)	mAP50-95 (%)	Parameters (M)	FLOPs (G)
YOLOv8n-Head	92.6	90.8	93.6	85.4	0.10	0.5
YOLOv11n-Head	94.5	92.4	95.3	87.8	0.08	0.5
DR-Head	**96.5**	**95.2**	**96.2**	**88.6**	**0.04**	**0.3**

**Table 3:** Hyperparameter tuning of λ in VetStar knowledge distillation. The results were obtained using a fixed random seed of 0. Bold indicates the best performance among the compared methods.

λ	Precision (%)	Recall (%)	mAP50 (%)	mAP50-95 (%)
1.0	96.3	94.7	96.4	**89.7**
1.1	95.7	94.5	96.5	88.7
1.2	95.8	96.2	96.9	88.9
1.3	**96.6**	**97.4**	**97.4**	89.5
1.4	95.4	95.4	96.7	88.4
1.5	96.2	94.6	96.8	88.4

**Table 4:** Performance comparison between VetStar and YOLO series models (v8-v11). The results were obtained using a fixed random seed of 0. Bold indicates the best performance among the compared methods.

Model	Precision (%)	Recall (%)	mAP50 (%)	mAP50-95 (%)	Parameters (M)	FLOPs (G)
YOLOv11-one head	95.6	**97.1**	97.4	90.9	2.30	5.1
VetStar	96.5	95.2	96.2	88.6	**0.04**	**0.3**
Distilled-VetStar	96.6	96.2	97.4	89.5	**0.04**	**0.3**
YOLOv8n	**97.3**	96.5	97	92.1	2.68	6.8
YOLOv9t	97.2	96.7	97.2	**92.2**	1.73	6.4
YOLOv10n	97.2	95.7	97.3	92	2.69	8.2
YOLOv11n	96.3	96.8	**97.8**	91.3	2.58	6.3

**Table 5:** Comparison of overall accuracy, class-wise recall, and class-wise precision of different models. The results were obtained using a fixed random seed of 0. Bold indicates the best performance among the compared methods.

Model	Positive_ Recall (%)	Negative_ Recall (%)	Positive_ Precision (%)	Negative_ Precision (%)	Accuracy (%)
YOLOv11-one head	**95.9**	98.3	91.9	99.3	97.8
VetStar	91.9	98.5	94	99	97.5
Distilled-VetStar	94.1	98.3	94	99.2	97.7
YOLOv8n	94.1	**98.9**	95	99.4	98.1
YOLOv9t	94.5	**98.9**	**95.1**	99.3	**98.2**
YOLOv10n	93	98.4	**95.1**	99.3	97.6
YOLOv11n	95.2	98.4	93.1	**99.5**	97.7

**Table 6:** Overview of the line elimination method (✓: visible line, ×: invisible line).

Quality Control Line (C)	Detection line (T)	Result
×	✓	invalid
×	×	invalid
✓	×	positive
✓	✓	negative

## Data Availability

The authors do not have permission to share the data used in this study.

## Abbreviations

The following abbreviations are used in this manuscript:

CGIA	Colloidal gold immunoassay.
LOD	Limit Of Detection.
RDT	Rapid diagnostic test.
SVM	Support vector machines.
CNN	Convolutional neural networks.
ROI	Region of Interest.
R-CNN	Region-based Convolutional Neural Network.
SSD	Single Shot MultiBox Detector.
YOLO	You Only Look Once.
API	Application Programming Interface.
UI	User Interface.
DR-head	Depthwise Separable–Reparameterization Detection Head.
BCKD	Bridging Cross-task Protocol Inconsistency Knowledge Distillation.
DW-Conv	Depthwise-separable Convolution.
FCs	Fully Connected Layers.
Rep-Conv	Reparameterization Convolution.
BCE	Binary Cross-Entropy.
IoU	Intersection-over-Union.
AMP	Mixed-precision training.
AP	Average Precision.
mAP	Mean Average Precision.
FLOPs	Floating Point Operations Per Second.
TP	True Positive.
FP	False Positive.
FN	False Negative.
TN	True Negative.
PPV	Positive Predictive Value.
NPV	Negative Predictive Value.

## Appendix A

Example of json file generated in software module:

To improve transparency, we provide 50 example images, which are available at: https://pan.baidu.com/clq/fyh (accessed on 7 September 2025).
